# A feasibility study using TomoDirect for craniospinal irradiation

**DOI:** 10.1120/jacmp.v14i5.4304

**Published:** 2013-09-06

**Authors:** Ulrich W. Langner, Janelle A. Molloy, John F. Gleason, Jonathan M. Feddock

**Affiliations:** ^1^ Department of Radiation Medicine Markey Cancer Center University of Kentucky Lexington KY USA

**Keywords:** TomoDirect, craniospinal irradiation

## Abstract

The feasibility of delivering craniospinal irradiation (CSI) with TomoDirect is investigated. A method is proposed to generate TomoDirect plans using standard three‐dimensional (3D) beam arrangements on Tomotherapy with junctioning of these fields to minimize hot or cold spots at the cranial/spinal junction. These plans are evaluated and compared to a helical Tomotherapy and a three‐dimensional conformal therapy (3D CRT) plan delivered on a conventional linear accelerator (linac) for CSI. The comparison shows that a TomoDirect plan with an overlap between the cranial and spinal fields might be preferable over Tomotherapy plans because of decreased low dose to large volumes of normal tissues outside of the planning target volume (PTV). Although the TomoDirect plans were not dosimetrically superior to a 3D CRT linac plan, the patient can be easily treated in the supine position, which is often more comfortable and efficient from an anesthesia standpoint. TomoDirect plans also have only one setup position which obviates the need for matching of fields and feathering of junctions, two issues encountered with conventional 3D CRT plans. TomoDirect plans can be delivered with comparable treatment times to conventional 3D plans and in shorter times than a Tomotherapy plan. In this paper, a method is proposed for creating TomoDirect craniospinal plans, and the dosimetric consequences for choosing different planning parameters are discussed.

PACS number: 87.55.D‐

## I. INTRODUCTION

Medulloblastoma is a highly malignant tumor and the second most common central nervous system neoplasm in children, accounting for approximately 15% of all pediatric brain tumors.^(^ ^1^ ^,^ ^2^ ^)^ The current standard of care consists of maximal safe resection of the tumor, followed by craniospinal axis irradiation (CSI) with concurrent and adjuvant chemotherapy.^(3)^ The most up‐to‐date treatment strategies result in a five‐year survival rate of approximately 80% for average‐risk and 70%‐75% for high‐risk patients, respectively.^(^ ^1^ ^,^ ^3^, ^4^, ^5^ ^)^ Despite these relatively favorable outcomes, children suffer long‐term effects from radiation therapy. The most notable include impaired neurocognitive development, hearing impairment, growth retardation, endocrine dysfunction, cataract formation, cardiomyopathy, impaired fertility, and second malignancies.^3^, ^4^, ^5^ Perhaps the most significant improvements in reducing late toxicities among survivors can be attributed to lowering of the CSI dose from 36 Gy to 23.4 Gy in average‐risk patients, as identified from the CCG A9961 trial^(3)^ (see http://clinicaltrials.gov/ct2/show/NCT00002875 for more detail). High‐risk patients continue to require 36 Gy. A recently closed trial (ACNS 0331) attempted to further lower the CSI dose to 18 Gy in order to improve toxicity profiles. However, as the results are not yet mature, patterns of care data suggest that a further reduction of dose to less than 23.4 Gy may be associated with higher failure rates.

The goal of CSI is to give a homogeneous radiation dose to the entire neuraxis. This is a technically challenging problem in radiotherapy planning and delivery because of the need to irradiate a large and complex target volume uniformly, while still reducing dose to organs at risk (OARs). The majority of the previously mentioned late effects are dose and volume related; therefore, more complex radiation delivery techniques, such as intensity‐modulated radiation therapy (IMRT), could be utilized to reduce OAR dose and improve the already narrow therapeutic ratio.^(^ ^2^ ^,^ ^6^, ^7^, ^8^, ^9^, ^10^, ^11^ ^)^ However, separate isocenters for cranial and spinal fields remain an unavoidable problem for CSI treatments on conventional linear accelerators (both for three‐dimensional conformal treatment (3D CRT) techniques, as well as IMRT techniques), such that matching of these fields and junction changes continue to be necessary. Field matching leads to dosimetric heterogeneity and uncertainty in the planning target volume (PTV), which is further complicated if an IMRT technique is used. For older children, two posterior spine fields are frequently required, necessitating additional junctions and planning complexity.

To simplify field matching during IMRT techniques, solutions were developed where the matching was done by incrementally increasing the dose for each field over a defined region, resulting in a “blending” of the dose, rather than abrupt changes.^(6)^ The major downside is that blending the junctions on a conventional linear accelerator (linac) between multiple spinal fields will require multiple IMRT plans, which is quite labor‐intensive. An alternative is Tomotherapy (Accuray Inc., Sunnyvale, CA), where the radiation is delivered to the complete neuraxis in a helical fashion with one setup point, thus obviating the need for field matching.^(^ ^2^ ^,^ ^7^, ^8^, ^9^, ^10^ ^)^ Tomotherapy also allows complex modulation of dose, which can provide a conformal dose to the target volume while minimizing high‐dose regions in normal tissue.

The feasibility of Tomotherapy to deliver CSI is well documented.^(^ ^8^ ^,^ ^10^ ^,^ ^12^ ^)^ However, concerns for increased secondary malignancies due to higher integral doses, and the potential impact of prolonged sedation in the setting of longer treatment times (which can be approximately five times longer than standard 3D techniques), limits the widespread use of Tomotherapy in children.^(^ ^2^ ^,^ ^6^, ^7^, ^8^, ^9^, ^10^, ^11^ ^)^

TomoDirect (Accuray Inc.) is a treatment option on a Tomotherapy unit that enables 3D CRT techniques to be used. Stationary gantry positions are utilized as the couch propagates through the gantry, with the multileaf collimator (MLC) continuously conforming to the PTV volume. Two modes are possible for TomoDirect, an IMRT mode in which modulation is manipulated during planning, and a 3D CRT mode in which the leaves conform to the PTV with limited additional modulation. The ability of Tomotherapy to treat the patient in a single continuous plan without the need to junction fields is retained, without significantly increasing the integral dose to the patient or prolonging the treatment time. This is done by generating a plan using the principles of a standard 3D CRT CSI treatment on a conventional linear accelerator (referred to hereafter as 3D CRT (linac)) to achieve the aforementioned benefits without significantly increasing the integral dose.

In this paper, we investigate the feasibility of TomoDirect treatments for CSI, using standard 3D CRT beam arrangements. The dosimetric consequences for choosing different planning parameters are also discussed. The plans generated will be compared to Tomotherapy and 3D CRT (linac) plans to examine the feasibility of a TomoDirect approach. This study was approved by an institutional review board.

## II. MATERIALS AND METHODS

### A. Patients

TomoDirect plans were retrospectively created for two previously treated patients in our clinic. The patients were selected sequentially from the most recent cases treated using Tomotherapy and 3D CRT (linac), respectively. Both children were four years old, one male and one female, and each was considered to have average risk disease following a gross total surgical resection and no involvement of the cerebrospinal fluid based on MRI and cytology. The decision to utilize the different treatment techniques was based on the preference of the treating radiation oncologist. The two patients were similar in terms of height and weight; patient 1 weighed 18 kg with a PTV of 52 cm in length, and patient 2 weighed 16 kg with a PTV of 51 cm in length. Both patients completed CSI to 23.4 Gy, followed by a posterior fossa boost to 55.8 Gy using standard fractionation. For the purposes of this paper, comparisons were only made between different approaches to delivering the CSI and not the boost.

### B. Simulation and planning

The planning CT was acquired through the entire neuraxis with 257 slices of 2.5 mm slice thickness for patient 1 and 227 slices of 2.5 mm slice thickness for patient 2. For Tomotherapy, the patient had a customized aquaplast head mask in the supine position and radiopaque BBs placed on the patient's head. The patient was straightened by placing skin marks and a radiopaque BB on the patient's chest. The patient was also placed in a vacloc bag (Civco, Orange City, IA) for immobilization. For the 3D CRT (linac) setup, the patient was simulated in the prone position on a table pad with a custom aquaplast head holder. Separate isocenters were placed at the time of simulation for both the cranial and spinal fields.

The CSI PTVs for both patients were drawn to include the entire vertebral body so as not to cause growth abnormalities and scoliosis, which can occur when treating partial vertebral bodies in children. The treatment plan characteristics for each of the plans are given in Table 1. Plan 1 is the Tomotherapy plan, which was delivered using a pitch of 0.43, a modulation factor of 2.4, and a field width of 2.5 cm. The plan was calculated so that 95% of the PTV is covered by the prescription dose. TomoDirect plans were then developed with three beams placed at 90° and 270° for the cranial fields and 180° for the spinal field with a field width of 5 cm. Different scenarios for these plans were investigated to optimize the parameter selection. Most of these scenarios produced similar results. Therefore only results for the scenarios with and without the overlapping field junctions are shown. Scenarios were tested with pitches other than 0.215 (the default pitch for a 5 cm field is 0.5), high and low modulation, and normal tissue homogeneity on and off. In the TomoDirect terminology, more and less compensation refers to the amount of modulation that is allowed when the plan is calculated. More modulation will result in a more homogeneous dose distribution in the PTV, but will require a longer treatment time. If normal tissue homogeneity is turned on, the planning software will attempt to reduce hot spots in normal tissue outside of the PTV. It must be noted that TomoDirect plans are equivalent to 3D CRT, and modulation and homogeneity is not comparable to that achieved with Tomotherapy IMRT plans.

**Table 1:** Treatment plan parameters for different the modalities used in this study. Plans 1–3 were planned for Tomotherapy and plan 4 for a 3D CRT on a conventional linac (Varian 21 EX). The total treatment field size in the craniocaudal direction was ~ 52 cm for patient 1 and ~ 51 cm for patient 2. See Eq. (1) for the equivalent MUs. Treatment times do not include imaging and setup time

*Patient*	*Plan*	*Technique*	*Treatment Position*	*Field Width (cm)*	*Pitch*	*Modulation Factor*	*Treatment Time (s)*	*Expected MUs*	*Equivalent MUs*
1	1	Tomotherapy	HFS	2.5	0.43	2.4	864.0	12568	604
1	2	3 beam Tomo Direct Overlap region used	HFS	5.0	0.215	High	377.6	5095	490
1	3	3 beam TomoDirect No overlap between cranial and spinal fields	HFS	5.0	0.215	High	389.9	5274	507
2	4	3D CRT (linac) SSD is 100 cm for spine	HFP	40.0	‐	‐	~420s	388	388

HFS = head first supine; HFP = head first prone

**Figure 1:**
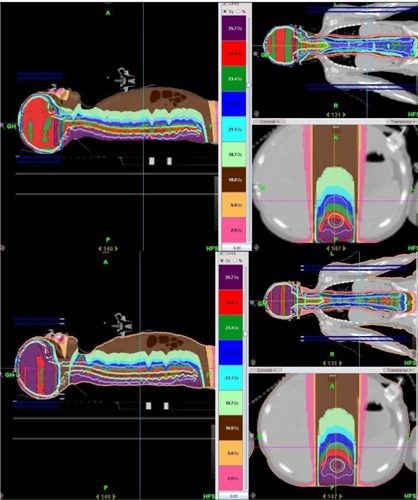
Dose distributions for TomoDirect plans with a 2.5 cm (half‐field width) overlap (plan 2 – top panel) and no overlap (plan 3 – bottom panel) between the cranial and spinal fields. Directional blocks were used to ensure only opposed lateral fields for the brain, a posterior field for the spine. The directional blocks for the brain and spine are indicated by the blue and gray dotted lines. Here 23.4 Gy represents 100%.

The TomoDirect plans were calculated so that 90% of the PTV was covered by the prescription dose. This is similar to what is done in the 3D CRT (linac) plan, where the spinal canal is covered by the 100% isodose line, but deeper parts of the vertebral body might be covered by lower isodose lines in order to reduce the maximum dose inside the PTV and minimize exit dose into anterior structures. Directional blocks were used to ensure that 90° and 270° beams were used only for the cranial fields (and not the entire neuraxis) and a posterior beam for the spinal field (see Fig. 1). Directional blocks mean that the beams cannot enter through a structure that is blocked before it encounters the PTV, but it can exit after encountering the PTV.

An overlap region was created in the TomoDirect plans (except plan 3) by terminating these blocks for a few slices in a junction region, in order to “merge” the fields as smoothly as possible. The length of this region was chosen so that the dose distribution of each field matches at ~ 50% isodose line. The effect of not using this overlap region is investigated in plan 3 (i.e., the cranial block starts on the CT slice immediately superior to the slice were the spinal block was terminated (see Fig. 1)). The effects of an increased pitch, of allowing less modulation and switching the normal tissue homogeneity off, and of increasing the source skin distance (SSD) were also investigated for the TomoDirect plans to show its effect on the plan quality. These plans produced similar results and results are, therefore, only discussed and not shown. A fine calculation grid (2 mm × 2 mm) was used for all the Tomotherapy and TomoDirect plans.

For the 3D CRT (linac) plan (delivered on a Varian 21 EX; Varian Medical Systems, Palo Alto, CA), a standard field‐in‐field technique was used with a 3 mm × 3 mm calculation grid: two standard opposed lateral cranial fields with the gantry at 90° and 270°, and a posterior spinal field with the gantry at 0°. Additional field‐in‐fields were added to deliver a more homogeneous dose to the brain and spinal canal. Due to the patient's size, the spine was feasibly treated in a single field, thereby requiring only one junction (with the cranial fields). A composite plan was calculated in which the junction was feathered after every 5 fractions by shifting it 5 mm inferiorly.

The equation used to calculate the equivalent Tomotherapy monitor units (MUs) is given by:

(1) $$\text{Equivalent MU}=\frac{\text{Total MU}}{\left(\text{Total field size in cranio}‐\text{caudal direction}/\text{field width}\right)}$$

The equivalent monitor units are used to give an indication on the amount of modulation, as well as the expected low dose to the patient. It normalizes the MUs so that it can be compared to 3D CRT plans.

The conformality of each plan was tested by calculating the ratio of the volume of the PTV receiving the prescription dose or higher $\left(V_{\text{Rx},\text{PTV}}\right)$ to the volume of the patient receiving the prescription dose or higher $\left(V_{\text{Rx},\text{patient}}\right)$,^(13)^ given by:

(2) $$\text{Dose}\;\text{conformality}=V_{\text{Rx},\text{PTV}}/V_{\text{Rx},\text{patient}}$$

The homogeneity of the dose distribution in the PTV was evaluated by calculating the ratio of the difference between the maximum and minimum doses inside the PTV to the prescription dose, given by:

(3) $$\text{Dose}\;{\text{homogeneity}=(D}_{2\%\text{PTV}}{{‐D}_{98\%\text{PTV}})/R}_{x}$$

Low‐dose volumes were assessed by recording the volumes of the patient receiving 2, 5, and 10 Gy, respectively, as well as the dose to 50% of the volume of each patient. The decision was made not to calculate integral doses because it has been shown before that different plans can give similar integral doses, yet the low‐dose volumes may be significantly different.^(^ ^12^ ^,^ ^13^ ^)^ Using $V_{2},V_{5},V_{10}$, and dose to 50% of patient volume were thought to be more meaningful measurements of low‐dose volume, as it pertains to concerns about second malignancy.

## III. RESULTS

Table 1 gives the different parameters that were used for each plan. Table 1 shows that the equivalent MUs are ~ 55% higher for the Tomotherapy plan (plan 1) compared to the 3D CRT (linac) plan (plan 4), while the MUs for the TomoDirect plans (plans 2 and 3) are on average ~ 29% higher than that of plan 4. For a TomoDirect plan where less compensation and no normal tissue homogeneity were used, the equivalent MUs were comparable to that of plan 4.

Table 2 shows the dose comparisons for different CSI plans. The contoured volume for patient 1 was 13,490 cc and a PTV volume of 1,871 cc (plans 1 to 3). For patient 2 (plan 4), the contoured patient volume was 10,083 cc and a PTV volume of 1,761 cc.

**Figure 2:**
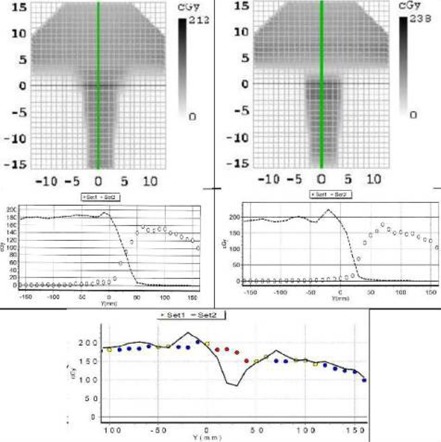
Fluences for plan 2 (set 1 ‐ left panels) and plan 3 (set 2 ‐ right panels) as measured with a MapCHECK2 diode array. The middle panels show the profiles along the line in the top panels to illustrate the contributions from cranial and spinal fields separately, while the bottom panels show the profiles from the composite plans of plan 2 (set 1) and plan 3 (set 2).

Figure 2 shows the overlap region which was used to match the cranial and spinal fields for the TomoDirect plans, as well as its dosimetric effect. Lateral directional blocks for the spinal field and a posterior directional block for the cranial fields were used to ensure that only opposed lateral fields for the brain and a posterior field for the spine are used. The blocks had a 2.5 cm (1 field width) overlap for the TomoDirect plans (except plan 3) between the cranial and spinal fields. Plan 3 was done with no overlap between these fields, but instead an abrupt change occurred from one slice to the next. In Fig. 2, the fluences and profiles in the cranio‐caudal direction, measured with a MapCHECK2 diode array (Sun Nuclear, Melbourne, FL) for plans 2 and 3, are shown.

**Figure 3:**
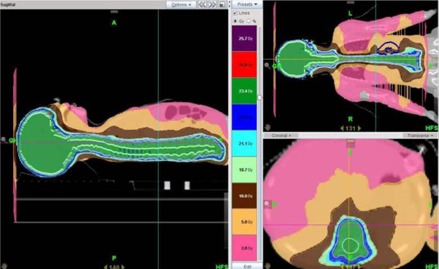
Dose distributions for a Tomotherapy plan with a field width of 2.5 cm (Plan 1 in Table 1). Here 23.4 Gy represents 100%.

**Figure 4:**
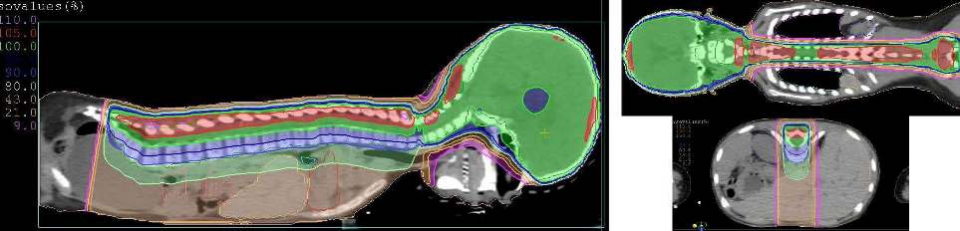
Dose distributions for a 3D CRT (linac) plan (plan 4 in Table 1). The spinal fields are setup to 100 SSD. Here 100% represents 23.4 Gy.

**Figure 5:**
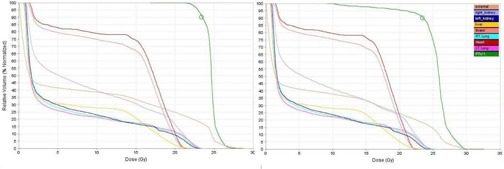
Dose‐volume histograms for the TomoDirect plans 2 (left panel) and 3 (right panel) in Table 1. Here 100% represents 23.4 Gy.

**Figure 6:**
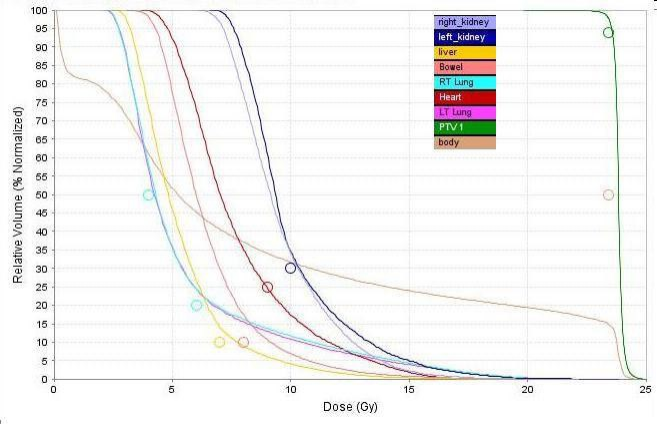
Dose‐volume histograms for the Tomotherapy plan (plan 1 in Table 1). Here 100% represents 23.4 Gy.

**Figure 7:**
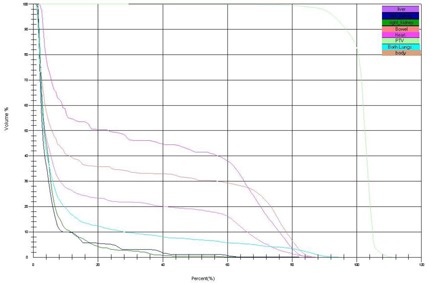
Dose‐volume histograms for a 3D CRT (linac) plan (plan 4 in Table 1). Here 100% represents 23.4 Gy.

Figures 3 and 4 demonstrate dose distributions resulting from plans 1 and 4. Figures 5, 6, and 7 show the dose‐volume histograms (DVHs) for plans 2 and 3 (Fig. 5), as opposed to those of plans 1 (Fig. 6) and 4 (Fig. 7).

**Table 2:** Dose comparisons for the different plans calculated in this study. Plan 1 was calculated using Tomotherapy, plans 2 and 3 using TomoDirect (3D CRT delivered on a Tomotherapy machine), and plan 4 used a 3D CRT (linac) plan (Varian 21 EX). Plans 1 to 3 are for patient 1 and plan 4 is for patient 2. For a description of the parameters used for each plan, see Table 1. The conformality is given by $V_{\text{RX},\text{PTV}}/V_{\text{RX},\text{patient}}$ and the dose homogeneity by $(D_{2\%\text{PTV}}\;-\;D_{98\%\text{PTV}})/R_{x}$

	*PTV*	*PTV*	*Dose*	*Heart*	*Kidney*	*Bowel*	*Lung*	*Patient*	*Spinal Cord*
*Plan*	$D_{98\%}\;(\text{Gy})$	$D_{2\%}\;(\text{Gy})$	*Homogeneity*	*Conformality*	$D_{5\%}\;(\text{Gy})$	$D_{5\%}\;(\text{Gy})$	*Max (Gy)*	$V_{20}\;(\%)$	$V_{5}\;(\%)$	$D_{50\%}\;(\text{Gy})$	$V_{2}\;(\%)$	$V_{5}\;(\%)$	$V_{10}\;(\%)$	*Max (Gy)*
1	23.4	24.3	0.04	0.84	13.4	15.0	22.8	0.4	35.8	5.27	78.6	51.2	31.6	24.4
2	22.4	26.4	0.17	0.69	20.4	21.8	22.1	11.3	27.9	1.40	45.5	41.5	38.6	26.9
3	13.8	29.2	0.66	0.67	21.5	23.2	23.2	13.5	28.6	1.49	45.7	41.2	37.8	30.6
4	20.8	25.0	0.18	0.84	18.4	5.2	20.4	2.9	15.7	0.98	42.6	38.6	36.0	25.5

## III. DISCUSSION

Different patients were used for the comparisons to simulate the preferred setup for each modality — a prone patient for the 3D CRT (linac) plan and a supine patient for the Tomotherapy plans. The same dose and fractionation were used, and the patients' ages, weights, lengths, and anatomical dimensions were similar. Imaging and setup time were not included in Table 1. As there is no light field available on Tomotherapy (here for both Tomotherapy and TomoDirect plans) for verification, two regions were scanned for registration, one in the brain and one in the spine. An average shift between these regions was then calculated and applied. The imaging time on Tomotherapy, including registration, took approximately 20 minutes, which is similar to the time it takes for portal imaging and checking match lines on a linac for each isocenter. However, the linac approach would not necessitate daily portal imaging, while the Tomotherapy approaches would likely require daily MVCT scans. Extra care should also be taken with Tomotherapy setup because, if the patient is not straight relative to the simulation CT, the spine might be completely missed inferiorly. The treatment time (without image guidance) for plan 1 was more than double that of any other plans. For the TomoDirect plans, the treatment times where similar to those achieved using the 3D CRT (linac) plan.

The equivalent MUs are ~ 55% higher for plan 1 compared to plan 4. This discrepancy is caused by the increased modulation and number of beam angles used during the Tomotherapy approach. The MUs for the TomoDirect plans are on average ~ 29% higher than for plan 4 because there is still some limited modulation occurring. For the TomoDirect plan, where normal tissue homogeneity was turned off and low compensation was selected, the MUs were equivalent to that of plan 4. This TomoDirect plan gave similar dosimetric results for a markedly lower treatment time and equivalent MUs compared to the other TomoDirect plans. The TomoDirect plans with compensation set to high were more conformal with higher treatment times.

Table 2 shows that the Tomotherapy plan had a superior dose homogeneity with a variation of only 4% of the prescription dose, compared to the other plans with variations ranging from 17%–66%. The dose homogeneity for the TomoDirect plans was similar to that of the 3D CRT (linac) plan, except for plan 3 where the abrupt transition of the cranial to spinal fields caused a large cold spot in the dose distribution in that region.

The conformality indices for plans 1 and 4 are surprisingly similar, although the homogeneity in the target is markedly smaller for plan 1. For the TomoDirect plans, the conformality indices are more than 15% smaller than for plans 1 and 4, which means that a larger volume of the patient received the $R_{x}$ dose. This is a result of the inverse square effect of the decreased source axis distance (SAD) on a Tomotherapy machine (from 100 cm SAD to 85 cm SAD), which will result in a higher dose to shallower regions because the percent depth dose (PDD) curve decreases faster. As a result, to get the prescription dose at the deepest point of the PTV, the shallower doses will increase.

Using the default pitch of 0.5 instead of 0.215 in the TomoDirect plans did not appear to have a marked effect on the dose distributions.

Maximum doses were less for the heart and liver using plan 1, although it was similar for all the plans regarding kidney and bowel dose. The maximum dose in the cord was also less with plan 1 (104%) compared to the other plans (~ 115%). Again the effect of the decreased SAD on the Tomothearpy unit is evident; the maximum cord dose is ~ 115% for the TomoDirect plans compared to 109% for plan 4. A TomoDirect plan with an increased SSD (i.e., moving the patient down inside the bore) did not markedly improve the cord or other OAR maximum doses.

The dose to 5% of the OAR volume was ~ 50% smaller for the heart, liver, kidneys, and bowel for plan 1 compared to the TomoDirect approaches. The largest advantage of Tomotherapy was in the lung $V_{20}$, which was ~ 3% compared to the other plans; however, the volume of lung receiving low dose $\left(V_{5}\right)$ was ~ 30% higher using Tomotherapy.

The increase in the low‐dose patient volume is further illustrated by the increase in $V_{2}$ of the patient from ~ 46% for the 3D CRT plans to 79% for Tomotherapy. $V_{5}$ was ~ 41% for the TomoDirect plans and 51% for Tomotherapy. However, the patient's $V_{10}$ for plan 1 is ~ 32%, compared to ~ 38% for the TomoDirect plans. The differences between the TomoDirect plans and the 3D CRT (linac) plan are mainly related to differences in the SADs between the machine geometries. Differences in patient anatomy also played a minor role in differences to OAR doses (a larger volume of the kidneys and bowel were in the field for patient 1). The patient $D_{50\%}$ increased by ~ 50% from plan 4 to the TomoDirect plans (i.e., from 0.98 Gy to ~ 1.48 Gy). The increase is even more remarkable when compared to that for plan 1, which gave a patient $D_{50\%}$ of 5.3 Gy (~ 0.40 Gy per fraction) (i.e., the 50% patient volume received at least five times more low dose with plan 1 compared to plan 4).

The 2.5 cm (half field width) overlap for the TomoDirect plans (except plan 3) between the cranial and spinal blocks ensured a smooth transition, as illustrated in the dose distributions shown in Figs. 1 and 2. From the bottom panel of Fig. 1, it can be seen that for the plan with no overlap (plan 3), a serious cold spot develops at the junction between these fields. This is further illustrated in Fig. 2 with the craniocaudal profiles of the fluences measured with the MapCHECK2 diode array. These profiles show the overlap for the cranial and spinal fields matched at approximately the 50% isodose line of each field for plan 2, while for plan 3 they are matched at approximately 25%. Note here that the measured doses are not the same for the cranial and spinal fields because the cranial fields were incident from the side of MapCHECK2 resulting in an under response of the diodes in this plane.

Figures 1, 3, and 4 compare the dose distributions from plans 2 and 3, and 1 and 4, respectively. From Fig. 1 and Fig. 4, it can be seen that the 105% and 110% isodose lines are extending much closer to the skin in the TomoDirect plan than for the 3D CRT (linac) plan because of the inverse square effect of the smaller SAD. The 2 Gy contributions are also spreading less out of the irradiated volume when compared to the Tomotherapy plan in Fig. 3. The more conformal and homogeneous dose distribution of the Tomotherapy plan is evident.

From the DVHs in Fig. 5 it is clear that the different TomoDirect plans give similar results. For the plan without an overlap between the cranial and spinal fields (plan 3), however, the cold spot inside the PTV is evident. A TomoDirect plan with an extended SSD (not shown) did not appear to have a large effect, although the left kidney DVH shifted to the left. From Fig. 7 it can be seen that the dose to small volumes of the OARs is less for plan 4 compared to the TomoDirect plans. From Fig. 6 it can be seen that plan 1 gives superior organ sparing when comparing higher doses. It can also be seen that the OARs in plan 1 receive larger low doses to a higher percentage of the OAR volumes than when using TomoDirect or 3D CRT (linac) plans. The Tomotherapy plan's excellent dose homogeneity results in a sharp DVH for the PTV.

## IV. CONCLUSIONS

From this study, it can be concluded that even though TomoDirect plans do not have the conformality and dose homogeneity in the PTV of a standard Tomotherapy plan, TomoDirect can generate plans comparable to 3D CRT (linac) plans. These plans might be preferable over Tomotherapy plans because of decreased low‐dose volume in normal tissues outside the PTV and shorter treatment times.

Although the TomoDirect plans were not dosimetrically superior to a 3D CRT (linac) plan, they provide some advantages. The patient can easily be treated in the supine position, which is often more comfortable and frequently necessary from an anesthesia standpoint. TomoDirect plans can be delivered in a comparable time to a 3D CRT (linac) plan. The most substantial benefit over 3D CRT linac plans may be that TomoDirect plans obviate the need for matching of fields and feathering of junctions.

This study shows that it is feasible to use TomoDirect for CSI if an overlap is used between the cranial and spinal fields to limit heterogeneity in the PTV. It may be especially useful if a treatment center only has a Tomotherapy unit but wants to avoid low dose to a large volume of the patient. Another possible use may be in children requiring endotracheal anesthesia, therefore necessitating treatment in the supine position.
